# A Systematic Review of the Benefits of Physical Exercise on Mental Health and Quality of Life in Patients With Substance Use Disorders

**DOI:** 10.7759/cureus.68781

**Published:** 2024-09-06

**Authors:** Farhana Nazmin, Eliseo Go, Moronkeji Fagbemi, Fatima Chowdhury, Natasha Kasulis, Saeed Sikandar, Saidur Rahman

**Affiliations:** 1 Psychiatry, BronxCare Health System, Bronx, USA; 2 Psychiatry and Behavioral Sciences, BronxCare Health System, Bronx, USA; 3 Internal Medicine and Addiction Medicine, BronxCare Health System, Bronx, USA; 4 Psychiatry, Middle Tennessee Mental Health Institute, Nashville, USA

**Keywords:** craving, mental health, physical exercise, quality of life, substance use disorders, systematic review

## Abstract

Physical exercise is increasingly recognized for its potential therapeutic effects in individuals with substance use disorders (SUDs), particularly in terms of relapse prevention, mental health improvement, and enhanced quality of life (QoL). This systematic review aimed to statistically summarize findings from numerous randomized controlled trials (RCTs) on the effects of physical exercise on mental health outcomes, QoL, abstinence-related outcomes, and cravings among SUD patients. A systematic search was conducted across PubMed, Web of Science, and Scopus databases, resulting in the inclusion of 15 studies, comprising RCTs, cohort studies, observational studies, and quasi-experimental designs. The review revealed a significant reduction in stress and depression, with a standardized mean difference (SMD) of 0.63, indicating a moderate effect size. Patients engaging in physical exercise reported a higher QoL across various domains, although the trend toward reduced cravings was not statistically significant, suggesting a need for further research in this area. The findings suggest that physical exercise can positively contribute to the treatment of SUD patients by enhancing mental health and QoL.

## Introduction and background

The fourth edition of the Diagnostic and Statistical Manual of Mental Disorders (DSM-IV) recognized "substance dependence" and "substance abuse" in two categories; however, the 2013 edition (DSM-V) combined these existing categories into one, which is "problematic substance use with a single category of Substance Use Disorder" (American Psychiatric Association, 2013), also called drug addiction. The World Drug Report (United Nations Office on Drugs and Crime, 2023) reported that in 2021, there were 297 million drug users worldwide and around 13.2% of them were injectable drug users. Currently, 39.5 million people are affected by substance use disorder (SUD), which is 45% more than at the beginning of the decade [1]. Substance use activates the brain's reward system, producing pleasure, which is a species of psychic joy whose nature is specific to the given substance. It can be produced by alcohol or caffeine or as a result of smoking cigarettes; synthetic cannabinoids, such as cannabis, lysergic acid diethylamide (LSD), and phencyclidine, are also capable of inducing euphoria. Methamphetamine is another stimulant that produces feelings of well-being. The most commonly abused stimulants today are methamphetamines, amphetamines, and caffeine. Inhalants like glue fumes from a paper bag or anesthetics as well as other volatile substances can produce a pleasing effect as can the poppy by-product opium, which includes heroin, barbiturates used as sleeping pills, and the pure form of gamma-hydroxybutyrate (GHB). SUD exhausts the dopamine pool in the brain, interferes with the linkage of dopamine and serotonin terminals, and engenders painful withdrawal symptoms, all of which generate anxiety or depression [2]. At the same time, it creates intense negative emotional states. SUD entails the brain cells making new connections to counteract the acute reinforcing effects of drugs [3], which leads to severe health problems and puts a heavy burden on the individual's life [4]. At present, the main treatments for SUD are pharmacologic, that is to say, using agonists to stimulate receptors (e.g., smoking substitutes to treat tobacco addiction or methadone and buprenorphine for opiate abuse). However, any excessive consumption of these treatments can also result in addiction to that medication [5].

Physical exercise has been found to work for patients receiving treatment for addictions. It can work as an adjunct intervention. Short-term aerobic exercise can also significantly improve the inhibitory deficits and drug cravings of methamphetamine users, and long-term physical exercise interventions help in aiding the treatment of substance addiction [6]. However, for the long-term physiological changes in people with SUDs to take effect, physical exercise must be practiced regularly and without interruption [7]. The theoretical and empirical research indicates that short-term and long-term aerobic exercise can reduce both drug cravings and abstinence urges with inhibitory control [8,9]. Carrying out such an intervention should facilitate the discharge of amines in their bodies [9]. The optimal duration for an adjunctive treatment intervention against SUD that combines aerobic exercises and drug regimens has yet to be determined, despite evidence from both theory and experience [10,11]. For the treatment interventions, aerobics of varying intensities have been exercised [10,11]. Although multiple repetitions of short-interval high-intensity interval training to observe a decrease in drug craving through Stroop cognitive tasks are used, both short-term and long-term results concerning the effects of aerobic exercise on SUD patients are currently lacking. Supplementary intervention effect studies that design a specific program of aerobic exercise and compare it with an anti-SUD drug control group are needed to determine whether these results are due to factors like background subjects or diagnostic procedures [12-14].

As per a study conducted by Brellenthin et al. in 2021, 30 minutes of exercise or rest can significantly improve overall feeling or mood during SUD treatment [14]. Exercise can also depress the negative emotions experienced during the withdrawal period. For example, tai chi has shown a significant positive effect on female and male heroin addicts [15]. Moreover, earlier studies found that aerobic exercise could decrease anxiety by increasing self-efficacy, because it reduces the risk of depression in SUD patients [16]. In individuals with an alcohol use disorder (AUD), physical activity improves both psychological well-being and emotional affective status by raising feelings of happiness and reducing guilt. It also decreased the likelihood that patients would develop depression [17]. Including physical exercise in the treatment of opioid use disorder can significantly reduce depression and anxiety [18]. A 2018 study by Zhu et al. found that 12 months of long-term tai chi exercise had an overall positive impact on the state of mind including depression in ATS patients otherwise known as amphetamine-type stimulant usage disorders [19].

The Brain Disease Model of Addiction points out that the risk of relapse to SUD is related to cognitive impairments [20]. Drug abuse, which only results in a marked reduction of prefrontal cortex and dorsolateral prefrontal cortex neurons in its later stages, will affect cognitive function related to drug-taking behaviors. Long-term withdrawal symptoms and post-withdrawal negative emotions (in terms of anxiety and depression) are important factors contributing to relapse [21]. It is, therefore, thought that improving cognitive function and reducing mental distress are effective treatment methods for patients with SUDs. Physical exercise has been found to influence brain cortical plasticity and improve cognitive function. It has also been found to eliminate attentional bias in SUD patients, improving cognitive abilities [22]. Cognitive functions are primarily composed of seven domains: visuospatial functioning, executive functioning, naming, memory, attention, language, and orientation [14,23,24]. It has been suggested that physical exercise may have a positive impact on behavioral performance related to these cognitive functions: inhibitory control, attentional bias, and working memory. Activities of corresponding brain cortical regions, including the prefrontal and dorsolateral prefrontal cortex, can also be enhanced according to their findings. Three months of tai chi training can significantly enhance overall cognitive abilities, including the maintenance of working memory and cognitive flexibility in SUD patients [13,25]. Twenty-four weeks of moderate-intensity aerobic physical exercise benefited their patients' perceptual and attentional functions. Zhu et al. conducted a study involving methamphetamine addict (MA) patients, which discovered that 12 weeks of moderate-intensity aerobic exercise as an addition to standard withdrawal treatment led to the significant return of a normal blood-brain barrier and brain neurons and increased inhibitory control [26].

Former studies have shown that physical exercise has a good effect on SUD's emotions and cognitive function, while different forms of intervention (e.g., type, duration, frequency, and intensity adjustments) were used meanwhile not identifying an optimal physical exercise intervention. Therefore, this study employed an approach for the most rational exercise regimens to further enhance the emotional and cognitive levels of people with SUD. This study is significant to the intervention research of SUD. By looking at the effects of an exercise intervention on emotions and cognition, enhancing our understanding of the psychological mechanisms involved in SUDs, we are then able to provide theoretical underpinnings plus practical guidance, for better implementation and better intervention measures.

## Review

Search strategy

**Figure 1:**
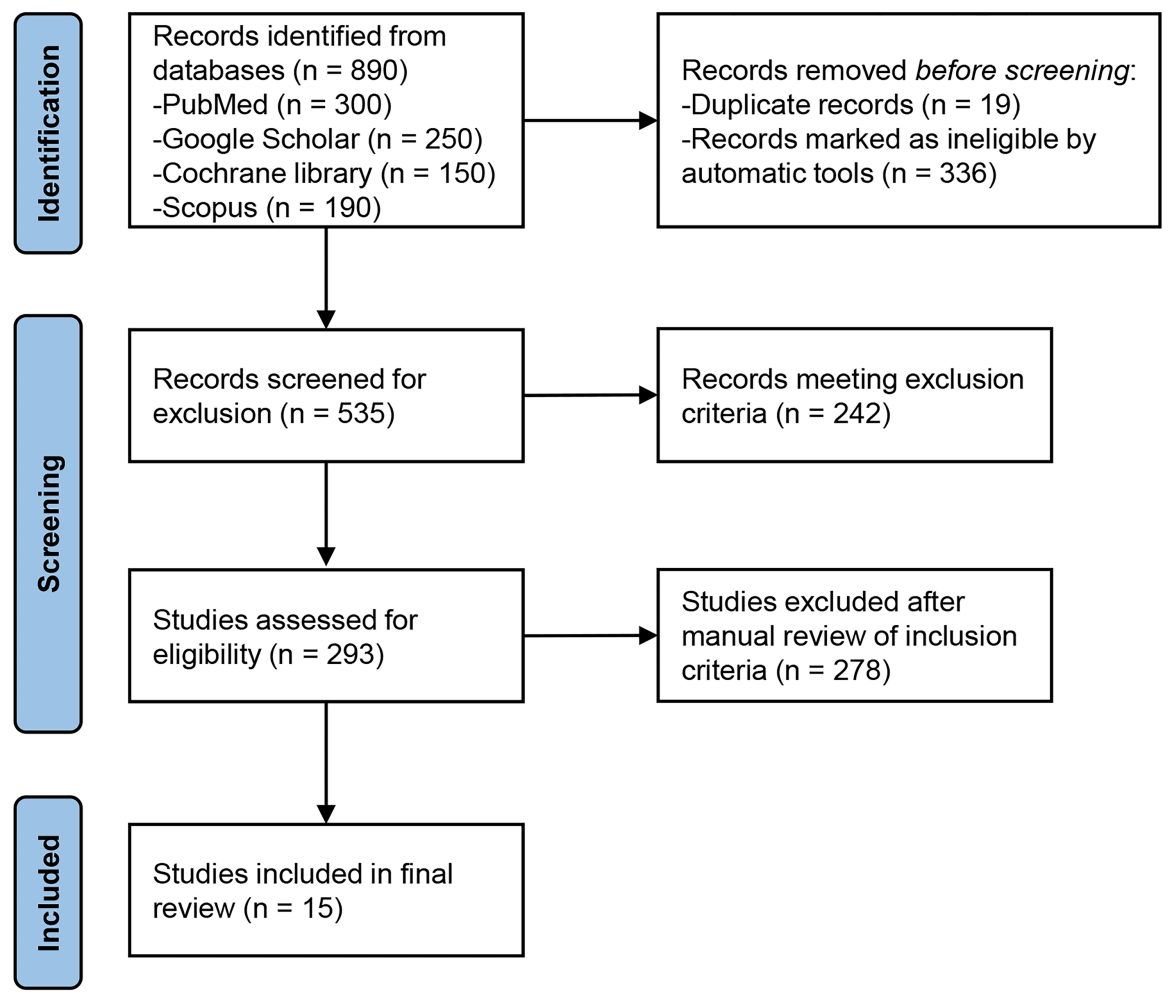
PRISMA flowchart showing included and excluded studies. PRISMA: Preferred Reporting Items for Systematic Reviews and Meta-Analyses

This systematic review was based on the Preferred Reporting Items for Systematic Reviews and Meta-Analyses (PRISMA) (Figure 1) guidelines [27]. The search terms included terms such as "exercise," "physical activity," "yoga," "substance use disorders," and "drug abuse" and specific substance names like "cocaine," "cannabis," and "alcohol."

Selection of studies

The selection criteria for this review included studies that evaluated the immediate- or long-term (≥2 weeks) benefits of exercise for patients diagnosed with alcohol or SUDs, as well as studies that employed body-mind exercises such as tai chi, yoga, or qigong (Table 1). Exclusion criteria included animal studies, research on patients under the age of 18, research exclusively focused on tobacco addiction, review studies, observational studies lacking physical activity programs, and case studies. A total of 890 studies were examined, and 875 of these studies were excluded from this study due to their failure to satisfy the set criteria. This study encompassed 15 studies that satisfied the criteria (Table 2).

**Table 1:** Inclusion and exclusion criteria. AUD: alcohol use disorder; SUD: substance use disorder

Criteria	Inclusion	Exclusion
Study population	Patients diagnosed with AUD and SUD	Studies involving animal subjects or patients under the age of 18
Intervention	Physical exercise or body and mind activities such as tai chi, yoga, or qigong	Studies exclusively analyzing tobacco addiction
Outcome measures	Analyzing the short-term (one session) or long-term (two weeks) impacts of physical activity on mental health issues, life satisfaction, abstinence, and craving	Studies not measuring relevant outcomes such as mental health, quality of life, abstinence, or craving
Study design	Experimental or quasi-experimental studies with a control group	Review articles, observational studies without a physical exercise program, case studies
Language	English-language publications	Studies published in languages other than English
Data availability	Sufficient data for systematic review or additional data obtainable from authors	Studies lacking relevant data for this study and where data could not be obtained from authors

**Table 2:** Summary and characteristics of included studies.

Study	Population/substance	Sample (exp+control)	Type of exercise program	Adherence to program (% attendance)	Measured variables	Main results
Gary and Guthrie, 1972 [28]	Liquor	20 (10+10)	3 weeks, 5 sessions/week of running 1 mile per day	-	Cardiovascular fitness, sleep quality	Improved cardiovascular fitness, reduced sleep problems
Frankel and Murphy, 1974 [29]	Liquor	214	11 weeks, aerobic and strength exercises, 5 sessions/week	-	Fitness, personality metrics	Enhanced physical fitness and some improved personality traits
Piorkowski and Axtell, 1976 [30]	Liquor	26 (14+12)	3 weeks, 5 sessions/week of circuit training	-	Stair climbing, heart rate	Improvement in physical fitness metrics
McKelvy et al., 1980 [31]	Liquor	48 (31+17)	5 weeks, 5 sessions/week of running 1.3 miles	-	Step tests, manual heart rate measurement	Improved resting and effort heart rate
Sinyor et al., 1982 [32]	Liquor	79 (58+12+9)	7 weeks, aerobic exercise and stretching, 5 sessions/week	-	Cardio-physiological and anthropometric measures	Increased abstinence rates, improved fitness
Palmer et al., 1995 [33]	Liquor	53 (27+26)	4.5 weeks, of aerobic exercise, 3 sessions/week	-	Anxiety, depression scales, self-concept, fitness tests	Reduced anxiety and depression, no change in physical fitness
Burling et al., 1992 [34]	Substance use disorder (varied substances)	95 (34+61)	3 weeks, 1 session/week of softball	-	Abstinence, sociodemographic data	Increased abstinence
Palmer et al., 1988 [35]	Substance use disorder (varied substances)	45	5 weeks, 3 sessions/week of supervised exercise	-	Depression scales, health status, fitness tests	Improved depression, no significant change in physical fitness
Shaffer et al., 1997 [36]	Substance use disorder in maintenance with methadone	59 (30+29)	20 weeks, yoga, 70 min/session	100%	Symptom checklist, addiction severity index	No significant differences between groups
Li et al., 2013 [15]	Morphinan opioid substance	86 (34+26+26)	11 days of qigong, 25-30 min/session	-	Urine analysis, anxiety scales, withdrawal symptoms	Improved withdrawal symptoms and anxiety levels
Khalsa et al., 2008 [37]	Substance use disorder (varied substances)	8	80 days of yoga	-	Perceived stress, behavior scale, quality of recovery index	Improved behavior, stress levels, and recovery quality
Weinstock et al., 2008 [38]	Substance use disorder (varied substances)	187 (45+142)	Participants chose at least one sport activity	-	Addiction severity, urine tests	Increased abstinence in those engaging in physical activities
Brown et al., 2010 [39]	Substance use disorder (varied substances)	16	10 weeks, moderate-intensity aerobic exercise	71%	Structured interviews, expired air analysis, fitness tests	Enhanced abstinence, improved cardiorespiratory fitness, no change in body composition
Chen et al., 2010 [40]	Substance use disorder (varied substances)	207 (126+81)	3 weeks, qigong or SMART relaxation techniques	92% (experimental), 78% (control)	Craving, sleep, anxiety, depression scales	Both groups showed improvement, with better adherence in the qigong group
Mamen et al., 2011 [41]	Substance use disorder (varied substances)	33	8.5 months (300 hours), aerobic exercise	-	Lactate measurements, heart rate, perceived exertion, VO2 max test	Significant improvements in aerobic power and lactate processing

Discussion

The reviewed studies (Table 2) demonstrate that incorporating regular physical activity into treatment regimens can result in significant improvements in both physical and mental health outcomes for patients. This discussion aims to delve deeper into the findings, explore their implications, and provide recommendations for future research.

Mental Health Improvements

One of the most striking outcomes reported across multiple studies is the reduction in psychological distress, particularly in stress, anxiety, and depression, among patients who participated in physical exercise programs. For example, Gary and Guthrie observed that a simple regimen of running 1 mile per day for four weeks led to notable improvements in cardiovascular health and reduced sleep problems in patients with AUDs [28]. Similarly, Palmer et al. found that aerobic exercise was effective in alleviating anxiety and depression among patients recovering from substance abuse [35].

Upgrades to Quality of Life (QoL)

Exercise also had a large effect on improving the QoL among SUD patients. Brown et al. found that stretched and aerobic exercise over a six-week program increased abstinence rates as well as physical fitness, thereby increasing the QoL [39]. This increased standard of living (QoL) is essential as it displays not simply bodily but additionally mental and social well-being, typically incredibly affected in individuals with SUDs.

The generalized benefits of physical activity, increased socialization, improvement in mental resiliency, and cardiovascular health, are likely what improve QoL. Rebuilding life skills and self-esteem are all things needed for long-term recovery and to prevent relapse experienced by those in sobriety from alcohol or substance dependence.

Exercise Program Heterogeneity and Patient Reactions

While there are obvious benefits with exercise, there is also a substantial problem concerning how exercise programs are implemented across research. The table includes a list of exercise modalities used (yoga, tai chi/qigong, and cardiovascular training/strength). For example, Shaffer et al. did not find significant differences between the groups receiving yoga and dynamic group psychotherapy [36]. Frankel and Murphy found objectively measurable physical benefits as well as various personality characteristics improved through aerobic and strength exercise [29].

This diversity highlights the need for future research that unravels the ideal exercise programs for the various populations with SUD. Other variables that might determine the patient's response to a given type of exercise include the substance of abuse, the duration of time that the patient has been addicted, and the physical status of the patient. The need for exercise programs that are individualized given these considerations can be envisaged as one way to increase the chances for therapeutic success.

Control of Cravings and Cognitive Function

Another possible research direction is the after-effects of exercise on cognitive function and desire control among individuals with SUDs. Several studies in this review, such as Menglu et al. and Zhao et al. [12,22], have shown that engaging in regular aerobic exercise is associated with improvements in higher-order cognitive functions, including working memory, inhibitory control, and attentional bias. This is particularly important because urges and cognitive deficits are major contributors to relapse in individuals with SUD.

Although the cognitive function benefits are clear, how they will be used in the regulation of cravings remains unclear. For example, some studies found a trend for a decrease in cravings, such as Li et al. with qigong interventions, while other studies found no discernible change [15]. This therefore shows that while taking part in physical exercise could facilitate the decrease of cravings at the highest levels, it may be useful in conjunction with other therapies, such as medication or possibly even behavioral therapy.

Benefits Over Time and Relapse Prevention

Long-term benefits of physical exercise in the prevention of relapse among individuals with SUDs were also investigated in several studies reviewed. For instance, Brown et al. assessed the effect of moderate-intensity aerobic exercise administered for 12 weeks on cardiorespiratory fitness and exercise adherence in participants maintaining abstinence [39]. Similarly, Weinstock et al. found that higher physical sporting activity participation was related to higher rates of abstinence during a contingency management program for SUDs [38].

Long-term recovery plans may include regular physical activity, management of withdrawal symptoms, and strategies to reduce the risk of relapse. Besides that, regular exercise is a planned activity that can take the place of substance-related behaviors. The constancy and routine of regular exercise may provide the stability that many recovering folks need to keep up their abstinence.

## Conclusions

The findings of this review highlight the need for further research to standardize the use of physical exercise in treating SUDs, focusing on identifying the optimal type, dose, and duration of exercise for various SUD populations. Future studies should also explore combining physical exercise with other treatment modalities, such as medication or cognitive-behavioral therapy, and conduct long-term follow-ups to evaluate its effectiveness in preventing relapse. Understanding the specific mechanisms through which exercise influences craving control and cognitive performance will be crucial for developing targeted interventions. Ultimately, this research could refine SUD rehabilitation programs and enhance long-term recovery outcomes through the strategic integration of physical activity.
